# Emerging opportunities to target inflammation: myocardial infarction and type 2 diabetes

**DOI:** 10.1093/cvr/cvae142

**Published:** 2024-07-19

**Authors:** Tafadzwa T J Kufazvinei, Jason Chai, Katherine A Boden, Keith M Channon, Robin P Choudhury

**Affiliations:** Division of Cardiovascular Medicine, Radcliffe Department of Medicine, University of Oxford, Oxford OX3 9DU, UK; Division of Cardiovascular Medicine, Radcliffe Department of Medicine, University of Oxford, Oxford OX3 9DU, UK; Division of Cardiovascular Medicine, Radcliffe Department of Medicine, University of Oxford, Oxford OX3 9DU, UK; Division of Cardiovascular Medicine, Radcliffe Department of Medicine, University of Oxford, Oxford OX3 9DU, UK; Division of Cardiovascular Medicine, Radcliffe Department of Medicine, University of Oxford, Oxford OX3 9DU, UK

**Keywords:** Diabetes, Myocardial infarction, Inflammation, Trained immunity

## Abstract

After myocardial infarction (MI), patients with type 2 diabetes have an increased rate of adverse outcomes, compared to patients without. Diabetes confers a 1.5–2-fold increase in early mortality and, importantly, this discrepancy has been consistent over recent decades, despite advances in treatment and overall survival. Certain assumptions have emerged to explain this increased risk, such as differences in infarct size or coronary artery disease severity. Here, we re-evaluate that evidence and show how contemporary analyses using state-of-the-art characterization tools suggest that the received wisdom tells an incomplete story. Simultaneously, epidemiological and mechanistic biological data suggest additional factors relating to processes of diabetes-related inflammation might play a prominent role. Inflammatory processes after MI mediate injury and repair and are thus a potential therapeutic target. Recent studies have shown how diabetes affects immune cell numbers and drives changes in the bone marrow, leading to pro-inflammatory gene expression and functional suppression of healing and repair. Here, we review and re-evaluate the evidence around adverse prognosis in patients with diabetes after MI, with emphasis on how targeting processes of inflammation presents unexplored, yet valuable opportunities to improve cardiovascular outcomes in this vulnerable patient group.

## 1. Introduction

The global prevalence of diabetes mellitus (DM) has increased markedly over the past 30 years, having more than doubled from around 200 million in 1990 to estimates of more than half a billion affected by 2025.^1^ This has important implications for acute myocardial infarction (MI), which is one of the most common causes of death amongst people with DM.^2^ Not only is the incidence of MI increased in people with DM, but the outcomes after MI are consistently worse. People with DM have higher rates of short and long-term mortality, re-infarction, heart failure (HF), cardiogenic shock, and arrhythmias post-MI.^3–8^ Treatment advances have greatly decreased the in-hospital mortality from MI, from ∼30% in the 1960s^9^ to <7% in the contemporary reperfusion era.^10^ However, despite the improvements in therapy and overall survival, people with DM show a persistent 1.5–2-fold increase in mortality compared to those without.^11^

This discrepancy in outcome for people with DM after MI is consistent across all management eras. In the era of conservative management, with no attempt at reperfusion therapies, the in-hospital mortality of people with DM following MI was 60.8%, twice that of people without DM.^12^ After the advent of coronary care units, a study of 832 people found that in the first month after acute MI, 20.2% of people without DM died as compared to 42.0% of people with DM.^13^ With reperfusion therapies, the GUSTO-1 study found that in people with segment (ST)-elevation myocardial infarction (STEMI), those with DM had a 30-day mortality of 10.5 vs. 6.2% in those without DM^14^ and in the GRACE Registry, those with DM and each of STEMI and non-ST-elevation myocardial infarction (NSTEMI) had higher in-hospital mortality (11.7 vs. 6.4% and 6.3 vs. 5.1, respectively).^15^

In the contemporary era of routine invasive arteriography and primary percutaneous coronary intervention (PCI), a single-centre study of 12 270 people (4388 with DM) from 2005 to 2013 demonstrated significantly higher 30-day and 1-year mortality rates in those with DM (13.5 vs. 4.3% and 25.7 vs. 12.4%, respectively).^16^ A sub-study of the PROSPECT II trial showed a two-fold increase in major adverse cardiovascular events (MACE) in people with DM.^17^ Similarly, the HORIZONS-AMI study found that at 30 days, the rates of death were higher in newly diagnosed and established DM (4.5 vs. 1.8%).^18^

A large majority of people with DM have type 2 diabetes (T2D); hence, most studies have been inadequately powered to study the impact of type 1 diabetes (T1D) on prognosis. However, in a nationwide Finnish population at 20 different hospitals, Kerola *et al.* investigated the case mortality of 1935 people with T1D compared to propensity-matched controls after MI from the years 2005 to 2018. People with T1D had a higher 30-day and 1-year mortality (12.8 vs. 8.5% and 24.3 vs. 16.8%, respectively) and this trend was consistent across different subgroups (with or without HF, with or without ST-elevation, with and without revascularisation).^19^ Therefore, despite different underlying pathophysiology, both T1D and T2D seem to negatively impact prognosis after MI.

Hyperglycaemia is a cardinal feature in both types of DM and elevated glucose levels on admission in people with acute MI is associated with an adverse prognosis. This has been well documented prior to reperfusion therapies,^20^ with the use of thrombolytic agents^21^ and in the contemporary era.^22^ A meta-analysis, including over 20 000 people with STEMI (23% with DM, but type not specified) treated with primary PCI revealed that admission hyperglycaemia (AH) was associated with higher mortality at both 30 days and long-term follow-up.^22^ It is important to note that AH is common in people without previously recognized DM, particularly in those with previous MI, multivessel coronary artery disease (CAD), and left-anterior descending infarcts.^22^ Despite these long-established observations, little progress has been made in answering the key questions surrounding hyperglycaemia in acute coronary syndromes (ACS), the chief of which is whether hyperglycaemia is a mediator or marker of adverse outcomes.^23^ The reader is directed to a recent comprehensive review of this topic.^24^

Most clinical studies focusing on MI prognosis do not distinguish between T1D and T2D. We can infer from their relative prevalence that most studies include predominantly people with T2D. In this review, unless specified otherwise, we are typically referring to T2D.

## 2. The effect of DM duration

In cardiovascular risk assessment, it is common to consider (in a binary fashion) whether a given person does or does not have DM. In fact, this type of nominal regard for DM potentially masks heterogeneity in important factors that include: (i) duration of DM, (ii) quality of glycaemic control, and (iii) treatments.

In T2D, duration has been associated with mortality risk. Baviera *et al*. analysed regional data of nearly 140 000 people hospitalized in Italy with STEMI or NSTEMI, between January 2010 and December 2019.^25^ People were divided according to DM status and people with DM were further divided into three groups according to the duration of disease: < 5 years, 5–10 years, and > 10 years. In their analysis, which included adjustment for age, in-hospital mortality increased with the duration of DM, with the highest risk in people affected for longer than 10 years when compared to people without DM (OR = 1.59, 95% confidence interval (CI) 1.50–1.69).^25^ This suggests that the adverse outcomes in T2D might not merely reflect the metabolic status at the time of the MI, but the accrual of consequential pathological features over time. Similarly, metanalysis (*n* = 25 studies and > 300 000 patients) of outcomes in relation to glycated haemoglobin (HbA1c) suggest that higher long-term glucose levels track with worse outcomes after ACS in T2D and, significantly, even in those without a nominal diagnosis of T2D.^26^

## 3. The effect of DM treatments

The effect that DM has on prognosis after MI might also be modified by specific treatments. In the GRACE Registry, STEMI patients with DM requiring insulin were at increased risk of death, HF, cardiogenic shock, and renal failure during the acute hospitalization period as compared to people on other DM treatments.^15^ Similarly, analysis of a pooled dataset of four randomized controlled trials including over 7000 participants with acute MI and DM showed that, when compared with people without DM, people with non-insulin-treated DM were at increased risk of cardiovascular death over a follow-up period of just under 2 years (hazard ratio (HR), 1.25; 95% CI, 1.13–1.38)^27^ but the magnitude of risk was even greater in people with insulin-treated DM (HR, 1.49; 95% CI, 1.31–1.69).^27^ In the SWEDEHEART Registry, people with DM on insulin monotherapy had an increased risk of the composite score of mortality, MI, stroke, and HF over a mean follow-up period of 3.4 years compared to people with DM treated with diet alone (HR, 1.32; 95% CI, 1.23–1.42).^28^ However, while these studies show a consistent pattern this may not necessarily be due to insulin therapy but might reflect confounding factors such as long-standing or poorly controlled DM that is not responsive to oral hypoglycaemic agents.

Metformin is commonly used as a first-line oral hypoglycaemic agent in T2D. In experimental models of MI, evidence suggests that metformin improves cardiovascular function and reduces infarct size.^29^ An observational study showed that metformin use was associated with lower peak creatine kinase-myocardial band and troponin levels^30^ but this same effect has not been shown elsewhere.^31^ In the SWEDEHEART Registry, people on metformin monotherapy had a lower risk of MACE as compared to people with DM treated with diet alone (HR, 0.92; 95% CI, 0.85–0.997).^28^ However, with no large randomized controlled trials to date, there is no definitive evidence to show that metformin has a causal role in improving outcomes post-MI. Similarly, no strong evidence exists concerning sulfonylurea use and its possible effects on MI prognosis. In an observational study of 188 people with MI and DM, Garratt *et al.* found that after multivariate adjustment, sulfonylurea use was an independent predictor of early mortality (odds ratio, 2.77; *P* = 0.017).^32^ However other studies have failed to show this association.^33–36^

While trials to investigate the effect of established hypoglycaemic agents on MI prognosis do not exist and are unlikely to be conducted, there is growing interest in the newer hypoglycaemic agents, particularly sodium-glucose cotransporter 2 inhibitors.

Sodium-glucose cotransporter 2 inhibitors provide cardiovascular outcome benefits in people with T2D. In the EMPA-REG OUTCOME trial, the use of once-daily empagliflozin compared with placebo was associated with a significant reduction in the incidence of death from cardiovascular causes (HR, 0.62; 95% CI, 0.47–0.77) and hospitalization for HF (HR, 0.68; 95% CI, 0.57–0.82) in participants with T2D at high cardiovascular risk regardless of reductions in HbA1c.^37^ Subsequently, randomized clinical trials of other SGLT2 inhibitors including canagliflozin,^38,39^ dapagliflozin^40^ and ertugliflozin^41^ have all been associated with a reduced risk of hospitalization for HF in people with T2D, demonstrating a consistent class-effect.

Furthermore, in the DAPA-HF trial, dapagliflozin was associated with a reduced risk of worsening HF (HR, 0.70; 95% CI, 0.59–0.83) and cardiovascular death (HR, 0.82; 95% CI, 0.69–0.98) in people with HF and an ejection fraction (EF) of 40% or less.^42^ As demonstrated in the DELIVER-TRIAL, dapagliflozin's beneficial effects extended to people with HF and a mildly reduced or preserved EF.^43^ Importantly, in both trials, these benefits were present in the presence or absence of DM.

The mechanisms by which SGLT2 inhibitors exert their cardiovascular effects are not yet understood but potential mechanisms have been reviewed elsewhere.^44,45^ SGLT2 inhibitors may affect inflammatory processes and the atherosclerotic plaque. In an observational study of people with T2D and multivessel non-obstructive coronary lesions undergoing PCI, SGLT2 inhibitor use was associated with higher values of minimum fibrous cap thickness (FCT), a measure of plaque vulnerability, and lower values of clinical measures of inflammation such as white blood cell count, high-sensitivity C-reactive protein (hs-CRP), interleukin-6 (IL-6), and tumour necrosis factor-alpha (TNFα).^46^

The large body of evidence demonstrating a consistent cardiovascular benefit raises the possibility that SGLT2 inhibitors might also be beneficial in the early period after MI.^47^ In an observational study of 377 individuals with T2D and acute MI undergoing PCI, current SGLT2 inhibitor use was associated with improved outcomes, regardless of glycaemic control.^48^ There is also evidence to suggest that in people with T2D, SGLT2 inhibitor use is associated with a lower risk of new-onset arrhythmia after MI.^49^

More definitively, randomized control trials investigating the role of SGLT2 inhibitors in the acute period after MI have recently been reported. In the EMpagliflozin in acute MYocardial infarction (EMMY) trial, 476 participants with acute MI (including 13% with T2D) were randomized to receive either once-daily empagliflozin or placebo for 26 weeks within 72 h of PCI. Empagliflozin use was associated with a significant reduction in mean N-terminal pro B-type natriuretic peptide levels and a significant improvement in left-ventricular EF, changes that were apparent in a matter of weeks.^50^ A *post hoc* analysis of the EMMY trial showed that empagliflozin did not significantly decrease systemic inflammatory markers such as IL-6, hs-CRP, neutrophil count, leucocyte count, and neutrophil/lymphocyte ratio when compared with placebo.^51^ In the DAPA-MI trial, 4017 people (without DM or HF) were enrolled to receive either 10 mg of dapagliflozin or a placebo in addition to standard-of-care therapy within 10 days of an MI.^52^ There was no difference in the rates of cardiovascular death or HF between the dapagliflozin and placebo groups. However, the trial had a low event rate, likely due to the exclusion of high-risk people and the short follow-up period; hence, it was inadequately powered to study these outcomes.^52^ The EMPACT-MI trial has recently reported a similar lack of effect of empagliflozin at a dose of 10 mg daily or placebo in people who had been hospitalized for acute MI, and were at risk of HF, and treated within 14 days after admission. A total of 3260 people were assigned to receive empagliflozin and 3262 to receive a placebo with a median follow-up of 17.9 months. Treatment with empagliflozin did not lead to a significantly lower risk of a first hospitalization for HF or death from any cause than placebo. Hospitalization for HF or death from any cause occurred in 267 people (8.2%) in the empagliflozin group and 298 people (9.1%) in the placebo group, with incidence rates of 5.9 and 6.6 events, respectively, per 100 patient-years (HR, 0.90; 95% CI, 0.76–1.06; *P* = 0.21).^53^

**Figure 1:**
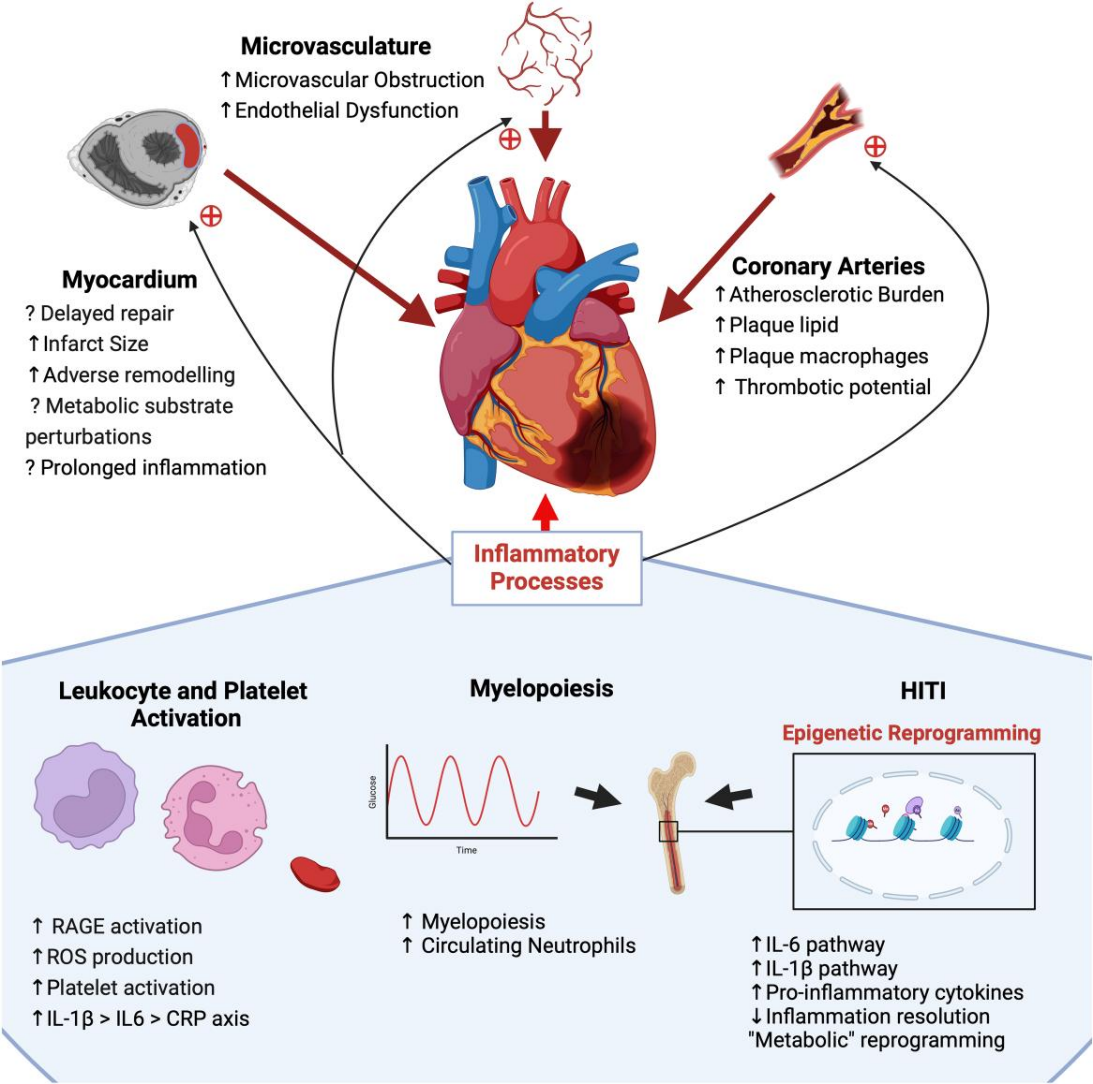
Potential for diabetes-associated adverse outcome after myocardial infarction. Adverse outcomes have been associated with features attached to the myocardium, microvasculature, and coronary arteries. Further differences in inflammatory processes including leukocyte and platelet activation, myelopoiesis, and HITI may contribute to this prognostic gap. CRP, C-reactive protein; HITI, hyperglycaemia-induced trained immunity; IL-1b, interleukin-1 beta; IL-6, interleukin-6; RAGE, receptor for advanced glycation end-products; ROS, reactive oxygen species.

Even as overall mortality has fallen, the proportionate increased risk associated with T2D has persisted, undiminished,^3^ emphasizing the need and urgency to understand, firstly, why people with T2D have worse outcomes specific to acute MI, despite contemporary treatment, and secondly how we can modify these outcomes effectively. In this review, we will re-examine some long-standing assumptions about the underlying mechanisms and evaluate emerging data that suggest new opportunities to target risk in this rapidly expanding population (*Figure 1*).

## 4. Potential causes of increased early mortality

In considering potential reasons for increased early mortality, it is useful to examine the mechanisms causing death after MI in people with DM. To our knowledge, no study has specifically investigated the mechanism of death in people with DM post-MI or asked whether there are different distributions of particular fatal manifestations compared with people without DM. While mortality is an unambiguous end point, determining the precise mechanism of death in people post-MI is challenging. Autopsy is undertaken in a small minority; consequently, data from death certificates are often inaccurate^54^ and seem unlikely to be illuminating in understanding the adverse risk of DM.

### 4.1 Infarct size and EF

Myocardial infarct size in DM has been plausibly proposed to contribute to the adverse prognosis.^55,56^ People with DM often have less severe or atypical MI symptoms^57^ that can lead to delays in seeking care, diagnosis, and treatment with the possible consequence of extending infarct size. Infarct size is strongly associated with mortality^58^ and limiting myocardial injury has been a key clinical priority driving the delivery of effective reperfusion therapies.

However, quantification of infarct size does not provide unambiguous support that people with DM sustain larger MI. Cardiac Magnetic Resonance (CMR) is the gold standard for non-invasive characterization of myocardial tissue,^59^ including determining infarct size post-MI. A CMR study of 92 people with MI (including 22 with DM) suggested that those with DM had larger infarct sizes as measured by late-gadolinium enhancement [mean % left ventricle (LV) scar, 25.6% vs. 15.8%],^55^ however the necessarily observational nature of the study and small sample size leave the significance of this finding uncertain; furthermore, subsequent CMR studies failed to show similar associations.^60,61^ Eitel *et al.*^60^ investigated the relationship between DM and infarct size in 411 STEMI patients undergoing primary PCI. Those with DM had a three-fold increased risk of MACE, even with similar infarct sizes (% LV scar 18.2 vs. 18.2%). Interestingly, AH was associated with a larger infarct size but this association was weaker in people with established DM.^60^ In another study of 792 people with STEMI across 8 centres in Germany, Reinstadler *et al.*^61^ found no significant difference in the infarct size or myocardial salvage index between people with or without DM. In addition to infarct size, reduced EF is also a predictor of mortality after MI,^62^ yet EF is comparable between those with and without DM.^55,56,61^ Therefore, it would seem that neither differences in infarct size nor EF adequately account for the impact of T2D on adverse prognosis after MI.

### 4.2 Atherosclerotic burden

Patients presenting with ACS typically undergo invasive coronary arteriography, triggered by the index acute presentation. Accordingly, assessment of the severity and extent of atherosclerosis in this context has often been attempted by systematic analysis of the clinically driven angiogram. However, it is also well-recognized that arteriography examines the vessel lumen which limits (i) the quantification of atherosclerosis that is contained within the wall of the artery and (ii) the determination of plaque composition, which in turn affects the plaque's biological properties, notably propensity to erosion or rupture. More detailed plaque characterization can be obtained using intravascular imaging with optical coherence tomography (OCT) and intravascular ultrasound (IVUS).

The prevalence of CAD is higher in people with DM,^63^ and it is widely thought that DM is associated with more severe and extensive CAD. In a comparative study between people with and without DM (*n* = 167, 47 with DM) presenting with ACS, Niccoli *et al.*^64^ described the more prevalent multivessel disease in the DM cohort (68 vs. 42%) with higher quantitative values of angiographically apparent CAD using multiple scoring systems including the Gensini score (18 vs. 11). Similarly, in a sub-study of the PROSPECT I trial, looking at the differences in plaque composition between people with and without DM presenting with ACS, people with DM had longer STs of CAD by IVUS (12.0 vs. 10.7 mm).^65^ In a different cohort, largely with stable angina, cardiac computerized tomography (CT) also demonstrated a higher number of coronary STs with a mixed plaque in people with DM (1.67 vs. 1.23) but when including all types of plaques (calcified, non-calcified, and mixed), there was no significant difference.^66^

However, the extent and severity of CAD can be difficult to describe and compare as the modality and method of quantification often varies between studies, and people recruited include those presenting with both stable angina and ACS. In addition, studies requiring invasive measurements using IVUS, OCT, and/or other invasive methods requiring instrumentation of the coronary arteries, may contain an inherent selection bias against those with complex and diffusely stenosed CAD. In the PROSPECT II trial, the mean SYNTAX scores in people with and without DM were not significantly different.^17^ Hence, although the pattern and severity of CAD have been implicated in the increased mortality in people with DM,^67^ this is not clearly the case.

PROSPECT II was a prospective natural history study in which the investigators used a combination of near infra-red spectroscopy and IVUS to identify features of non-obstructive plaques that are prone to cause future events.^68^ The study included 898 people who had a recent MI from 16 different hospitals across Scandinavia, between 2014 and 2017. The investigators found that both high lipid content (OR 3.80, 95% CI 1.87–7.70, *P* = 0.0002) and large plaque burden (OR 5.37, 95% CI 2.42–11.89, *P* < 0.0001) were independent predictors of non-culprit lesion MACE (defined as the composite of cardiac death, MI, unstable angina, or progressive angina).^68^ In a sub-study of PROSPECT II, people were stratified according to DM status. Despite contemporary stent technology and pharmacotherapy, the MACE rate was doubled in people with DM (OR 1.94 (95% CI, 1.14–3.30), driven mostly by spontaneous MI in non-culprit lesions and, to a lesser extent, restenosis in culprit lesions. In contrast, MACE in people without DM was driven predominantly by progressive angina. DM was an independent predictor of non-culprit lesion MACE (OR 2.47, CI 1.21–5.04), but did not seem to affect MACE in culprit lesions. Moreover, plaque characteristics (lipid content and plaque burden) in culprit and non-culprit lesions were similar in people with and without DM^17^, and there were no differences in baseline and residual SYNTAX scores.

Interrogating coronary arteries with OCT, in 250 people presenting with ACS, Sugiyama *et al*., reported (i) a higher prevalence of lipid-rich plaque and macrophage accumulation in the culprit vessel and (ii) greater maximal lipid arc, thinner FCT and a higher prevalence of thin-fibrous cap atheroma (TCFA) in the non-culprit vessel, in people with DM compared to those without.^69^ These findings suggest that, at the time of ACS, people with DM have more extensive CAD and more ‘vulnerable’ plaque. However, as highlighted by Ali *et al.*^70^, the determination of ‘vulnerable’ plaque by OCT is limited by modest interobserver agreement for cap thickness and lipid arc measurements compounded by the extremely low per-lesion event rates in people with TCFA.

While the burden of CAD at the time of MI may be greater in people with DM, this does not necessarily account for the impact of DM on prognosis after MI. In the GUSTO Angiographic Trial, Woodfield *et al.*^71^ compared baseline angiographic differences in 2431 STEMI patients randomized to four different thrombolytic regimens and found that people with DM had a greater frequency of multivessel disease and significantly smaller reference diameters in the infarct-related artery, but after adjustment for multivessel disease and 90-min thrombolysis in myocardial infarction (TIMI) flow, DM remained an independent predictor of 30-day mortality. While this may suggest that the burden of CAD does not account for all of the adverse prognosis, it is worth noting that this study used a fairly crude measure—the presence or absence of multivessel disease—and not more informative metrics such as the SYNTAX or Gensini score. Neither did this study investigate the composition of the coronary plaque.

Cumulatively, these studies suggest that differences in plaque extent, morphology, and lesion complexity as quantified and described by current methods such as invasive angiography, intravascular imaging, or cardiac CT are unlikely, on their own, to be sufficient to explain the increased rate of MACE in people with DM. In particular, the findings from the PROSPECT II trial sub-study suggest that additional pathophysiological mechanisms in people with DM are not dependent on plaque extent or morphology, but somehow extend their influence to cause acute MI in lesions that are remote from the index culprit lesion.

### 4.3 Myocardial perfusion and coronary microvascular dysfunction

Effective, timely reperfusion is a well-established determinant of outcomes in acute MI and requires both patency of the epicardial vessel and competence of the microvasculature. Even where primary PCI restores the former, coronary microvascular dysfunction (CMD) can delay or prevent reperfusion leading to persistent ischaemia and infarction. The coronary microcirculation consists of smaller arteries and arterioles <500 μm in diameter and is the main site for regulating myocardial perfusion.^72^ Although these vessels are not visible by current imaging modalities including invasive arteriography, their function can be assessed by physiological measurements. An abnormal coronary flow reserve (CFR) or equivalent indices such as myocardial flow reserve (MFR), which measure the ratio of myocardial blood flow at stress or maximal hyperaemia to that during rest provides a quantitative definition of CMD in the absence of epicardial stenoses.

Importantly, CMD impacts prognosis after MI; Kelshiker *et al.*^73^ have shown in their systematic review of CFR and cardiovascular outcomes that an abnormal CFR is a predictor of MACE, in both acute and chronic coronary syndromes. Studies specifically comparing people with and without DM have been few and mostly in the context of people with stable symptoms and not in acute MI.

In a single-centre study using Positron Emission Tomography in people with and without DM (*n* = 6019), an abnormal MFR was more prevalent in people with DM but both factors were independent predictors of MACE.^74^ Similarly, in a study using CMR to measure CFR by determining coronary sinus flow at rest and stress, in people with DM (*n* = 309), CFR < 2 was a predictor of MACE.^75^ These data have been replicated in invasive angiographic measures to determine CFR and also microcirculatory resistance specific indices such as Index of Microcirculatory Resistance, Hyperaemic Microvascular Resistance, and Microvascular Resistance Reserve,^76–79^ demonstrating that people with DM, mostly in the context of stable angina, are more likely to have a lower CFR (<2) and although less consistent, a high microcirculatory resistance index. Interestingly, CMD measured by stress echocardiography, prior to evidence of significant epicardial stenosis in a study of 144 people with T2D predicted death and non-fatal MI.^80^

Microvascular obstruction (MVO) in people with DM is also more prevalent and could plausibly contribute to excess mortality; a pooled analysis of STEMI patients from seven primary PCI randomized clinical trials found that MVO measured within 7 days of the acute event was strongly associated with mortality and hospitalization for HF.^81^ Following PCI, people with DM more frequently have incomplete ST resolution,^82^ reduced myocardial blush grade,^83^ and MVO as measured by CMR.^81,82,84^ It may be expected that MVO affects prognosis by increasing infarct size, but MVO is an independent predictor of adverse outcomes, even after adjustment for infarct size.^81^

Both invasive and non-invasive methods have shown that people with DM tend to have CMD, as measured by CFR which is an independent predictor of MACE, and are more likely to have MVO following STEMI. In the context of poorer outcomes in people with DM following MI, these findings could reflect (i) preceding coronary microvascular disease that impairs reperfusion or repair and/or (ii) aggravated functional microvascular dysfunction or obstruction during AMI that impairs myocardial reperfusion.

### 4.4 Diabetic cardiomyopathy

DM confers a two- to four-fold increased risk of HF^85,86^ and this is commonly attributed to ‘diabetic cardiomyopathy', which refers to abnormal myocardial structure and function in the absence of CAD, hypertension, and significant valvular disease in individuals with DM.^87^ An increased risk of HF is also apparent after MI, for example in the SWEDEHEART registry, where DM increased the risk of post-MI HF by 30%.^88^

In the diabetic heart, structural changes include cardiac fibrosis, cardiac hypertrophy/ remodelling, and microvascular dysfunction.^89^ A CMR study revealed that out of 107 people with DM with no clinical evidence of a previous MI, 30 (28%), had a myocardial scar as evidenced by late-gadolinium enhancement.^90^ Although the type of DM was not specified, the average age of the participants (59 ± 13) suggests that most were likely to have T2D.^90^ The high frequency of myocardial scar in this cohort suggests that either people with T2D have silent infarcts or that T2D independently causes fibrosis. Various mechanisms including fibroblast proliferation, neurohumoral activation, pro-inflammatory cytokine production, oxidative stress, increased myocardial transforming growth factor beta expression, and activation of the advanced glycation end products (AGE)/receptor for AGE (RAGE) axis may contribute to fibrosis in DM, as reviewed in detail elsewhere.^91^ Cardiac remodelling is often observed in people with DM^92,93^ and is most likely under-detected because only a few patients with DM undergo CMR imaging.

Hence, potentially diverse manifestations of a diabetic cardiomyopathy may explain why people with DM are more susceptible to the effects of an MI, either by impacting the infarcted area itself or through adverse effects on the remote myocardium, by impairing its ability to compensate for the damage to the infarcted muscle.

### 4.5 The prognostic effect of sex

Numerous studies have demonstrated that following MI, females tend to experience worse outcomes compared to males, including higher rates of hospitalization,^94^ HF^88,95^ short-term,^96–100^ and long-term mortality.^101,102^ However, this prognostic gap tends to diminish or disappear when confounding variables including age, risk factors, and co-morbidities are considered.^96,99,102,103^ Furthermore, females are less likely to receive guideline-approved therapies after MI in a timely manner,^98–100^ suggesting that this prognostic gap is not wholly explained by biological sex differences.

Female patients with DM may represent an extremely vulnerable patient group. An observational study investigated the rates of death and MI at 1 year among 17 154 individuals undergoing PCI, stratified by sex and DM status. Female patients with DM were at the highest risk of death and MI. Remarkably, female patients without DM exhibited a similar risk of experiencing adverse events as compared to male patients with DM. These findings persisted after multivariate adjustment.^104^ Evidence suggests that young females with DM are at particularly high-risk of long-term mortality after MI.^101,105^ However, with regards to short-term mortality, a previous study showed that amongst 48 878 individuals between the ages of 18 and 45 with DM and acute MI, females with DM were not at higher risk of in-hospital mortality compared to men after adjusting for confounders.^106^ Females are underrepresented in clinical studies and many knowledge gaps remain in understanding the influence of sex on MI prognosis, including its interaction with DM.

## 5. Emerging possibilities to identify and target inflammation

In the preceding sections, we have attempted to evaluate the contributions to adverse prognosis in T2D from clinically accessible assessments after MI. The situation is complex with potential contributions from inter-related factors, the individual contributions of which are difficult to tease out.

Furthermore, a large body of evidence points towards differential cellular and molecular responses in the context of DM. Some of these, such as oxidative stress, angiogenesis and altered cellular energetics are well-described and we will recap them briefly. We will focus in detail on emerging processes of inflammation and notably the direct effects of DM on immune cell function and their potential to unlock new possibilities for patient characterization and directed treatments.

After MI, excessive generation of reactive oxygen species (ROS) plays a crucial role in mediating ischaemic/reperfusion injury by directly damaging DNA, lipids, proteins, and mitochondria leading to increased cardiomyocyte death.^107^ ROS can also trigger inflammatory processes, for example by activating the nucleotide-binding oligomerization domain, leucine-rich repeat- and pyrin domain-containing protein 3 (NLRP3) inflammasome,^108^ the Janus kinase/signal transducer and activator of transcription (JAK-STAT) pathway^109^ and the nuclear factor kappa B (NF-κΒ) pathway.^110^ Patients with DM exhibit heightened NADPH oxidase activity and impaired endothelial nitric oxide synthase function in their blood vessels, leading to elevated production of ROS.^111^ Elevated levels of ROS are also observed in the myocardium of people with DM.^112^

Angiogenesis plays a crucial role in facilitating the repair of the injured myocardium by providing oxygen and nutrients to limit ischaemic damage.^113^ New vessel formation is required for the recruitment of leucocytes—essential mediators of injury and repair.^113^ Hypoxia-inducible factor 1-alpha (HIF1-α) serves as a pivotal regulator of angiogenesis and increased expression of HIF1-α in cardiomyocytes has been shown to enhance cardiac function and reduce infarct size in a mouse model of MI.^114^ It is well-established that hyperglycaemia impairs the stabilization of HIF1-α in endothelial cells.^115^ It has also been previously shown that ventricular biopsies of people with T2D exhibit decreased expression of HIF1-α and vascular endothelial growth factor when compared to people without DM.^116^ Other pathways relating to novel angiogenic proteins,^117^ defective endothelial progenitor cells,^118^ and vascular nitric oxide resistance^119^ may all contribute to impair angiogenesis in people with T2D post-MI. The reader is directed to reviews of this topic.^120,121^

Altered myocardial energetics post-MI may impact prognosis. Normally the heart uses a diverse range of substrates including free fatty acids, glucose, amino acids, and ketones to generate adenosine triphosphate.^122^ In the ischaemic heart, glucose is preferentially used as a substrate. However, in T2D, the heart ceases to be metabolically flexible. Hyperinsulinemia and insulin resistance decrease glucose utilization and increase free fatty acid intake by the myocardium leading to the accumulation of toxic lipid metabolites and ROS and eventually myocardial dysfunction.^122^

However, additional previously overlooked factors relating to processes of inflammation, appear increasingly relevant and plausible. Circulating markers of inflammation, most notably high-sensitivity C-reactive protein, have been associated with adverse outcomes in a variety of clinical contexts.^123–126^ In the CARE trial,^127^ evidence of systemic inflammation, as measured by elevated CRP and serum amyloid A (SAA), was associated with an increased risk of recurrent coronary events after MI, but with a higher proportion of events in people with T2D. Indeed CRP and SAA have long been recognized to be higher in people with T2D.^128^ More recently, a collaborative analysis of three randomized clinical trials by Ridker *et al.*^129^ investigated the association between cardiovascular events and death with each of low-density lipoprotein (LDL)-cholesterol and hs-CRP, in people in whom LDL-cholesterol had been intensively lowered, achieving contemporary standards. Amongst 31 245 people from three randomized clinical trials (PROMINENT, REDUCE-IT, and STRENGTH) elevated hs-CRP showed a stronger association with future events than did ‘on-treatment’ LDL-cholesterol.^129^ Importantly, the majority of people (76%) in this analysis had T2D, reinforcing the notion that processes of inflammation are important in this population.

Together, these studies suggest that processes of inflammation might drive worse outcomes in people with DM, who present with CAD. However, the use of the blanket term ‘*inflammation*’ is mechanistically uninformative. CRP, which is synthesized and released by the liver, represents the integrated downstream effects of several post-infarct-related inflammatory processes and is not sufficiently specific to describe complex molecular and cellular inflammatory processes.^130^ Functionally relevant information on specific molecular pathway activation, stage of involvement of complementary (or competing) cell types, status of acute inflammation vs. resolution, and even the precise loci of inflammation are currently not well-defined in MI. As a result, the optimal timing and nature of interventions to target potentially tractable processes of inflammation are unknown. Furthermore, targeting processes of inflammation requires consideration of sex differences in the immune response to MI.^131^ Hence, we will direct our focus to the processes of inflammation that are mechanistically important, therapeutically tractable, and subject to potential perturbation in people with DM.

## 6. Inflammation-related processes after MI

MI triggers local and systemic inflammatory processes. In the myocardium, the post-infarct inflammatory response can be thought of in three phases: the alarm phase, the leucocyte mobilization phase, and the resolution phase.^132^

In the alarm phase, dead cardiomyocytes and other cells release signals known as damage-associated molecular patterns (DAMPs) which include high mobility group protein B1,^133^ heat-shock proteins,^134^ S100A8/A9,^135^ and fibronectin.^136^ DAMPs bind to pattern-recognition receptors such as Toll-like receptors, nucleotide oligomerization domain–like receptors, and RAGE to activate innate immune pathways.^137^ Pro-inflammatory signalling pathways that can be activated within innate immune cells include, but are not limited to (NLRP3)/IL-1β,^138^ target lesson revascularization-4/NFκB,^139^ and the JAK-STAT signalling pathway^140^

In the leucocyte mobilization phase, leucocytes rapidly infiltrate the ischaemic myocardium.^141^ Neutrophils are the first cells to respond^142^ followed by pro-inflammatory monocytes^143^ (which subsequently differentiate into macrophages) to clear dead cardiomyocytes and other necrotic tissue. We have previously shown how endothelial cell–derived extracellular vesicles mobilize neutrophils and monocytes to the infarcted myocardium^144,145^ and how the transcriptomes of these cells are altered in the blood prior to their recruitment to the injured myocardium.^144,146^ Acute MI also mobilizes leucocytes to the myocardium remote to the ischaemic area.^147^

In the resolution phase, pro-inflammatory processes are suppressed. Macrophages transition to an anti-inflammatory, pro-resolving (M2) phenotype, and neutrophils are essential in mediating this process.^148^ In this phase, macrophage function switches from phagocytosis, proteolysis, and extracellular matrix degradation to angiogenesis and granulation tissue formation.^149^

Systemically, MI increases plasma concentrations of the pro-inflammatory cytokines TNF-α^150^ and IL-6.^151^ It is also worth noting that experimental MI accelerates atherosclerosis by increasing the recruitment of myeloid cells to the plaque.^152^

### 6.1 Potential therapeutic targets

Processes of inflammation are potential therapeutic targets. In experimental models of MI, inhibiting the recruitment of pro-inflammatory monocytes to the injured myocardium reduces infarct size.^153–155^ This has led to the notion that an exaggerated or prolonged pro-inflammatory response exacerbates injury and minimizes repair, leading to adverse ventricular remodelling.^149,156^ However, non-targeted inhibition post-MI can be detrimental. Corticosteroids and nonsteroidal anti-inflammatories haven been previously shown to be associated with ventricular wall rupture after MI.^157,158^ As previously highlighted, macrophages transition to become agents of healing, and it is understandable that non-targeted inhibition impairs healing/repair.

Targeted inhibition of inflammatory pathways in the context of CAD has shown promising results in recent years. The CANTOS trial in 2017 demonstrated a proof-of-concept that targeting an inflammatory pathway; i.e. IL-1β with a monoclonal antibody, canakinumab, can modify disease outcomes in people with known CAD and hs-CRP of at least 2 mg/L,^159^ although the effect size was modest. Subsequently, IL-6, a more downstream cytokine, is currently being investigated as a potential therapeutic target. In ASSAIL-MI, a Phase 2 randomized clinical trial of 199 individuals, targeting IL-6 with tocilizumab significantly increased the myocardial salvage index in patients with STEMI presenting for primary PCI within 6 h of chest pain.^160^ In RESCUE, a Phase 2 randomized clinical trial, Ziltivekimab, a monoclonal antibody targeting IL-6, demonstrated a significant reduction in CRP levels, without affecting the level of cholesterol, in a cohort of people with impaired renal function [estimated glomerular filtration rate (eGFR) 10–60 mL/min per 1.73 m^2^] and hs-CRP of at least 2 mg/L.^161^ A Phase 3 trial, ZEUS, investigating clinical outcomes has recently completed recruitment. A potentially even more relevant Phase 3 trial, ARTEMIS, will examine the effects of ziltivekimab started as early as possible after an invasive procedure, and latest within 36 h of hospitalization for STEMI, and latest within 48 h of hospitalization (time 0) for NSTEMI.

Both IL-1β and IL-6 are active cytokines that are implicated in causal pathways of CAD and its complications and are therefore potential therapeutic targets. This is in contrast to C-reactive protein, which is an acute phase protein made in the liver in response to IL-6, but without a causative role in disease pathogenesis; it is merely a marker. Importantly, targeted inhibition of the causal pathways should provide a better mechanistic understanding of inflammatory processes that drive the progression of atherosclerosis and help characterize and define patient groups who are likely to benefit most. In the following sections, we will highlight how emerging data suggest that processes of inflammation are of particular relevance to people with T2D, providing new opportunities for diagnostic stratification/characterization, monitoring, and treatment.

## 7. DM and processes of inflammation

There is a growing interest in understanding how metabolic disorders, such as DM, affect immune cell function. Much remains to be understood, but current evidence suggests important possibilities in relation to how DM could aggravate processes of inflammation after MI, and how this could mediate adverse outcomes.

**Figure 2:**
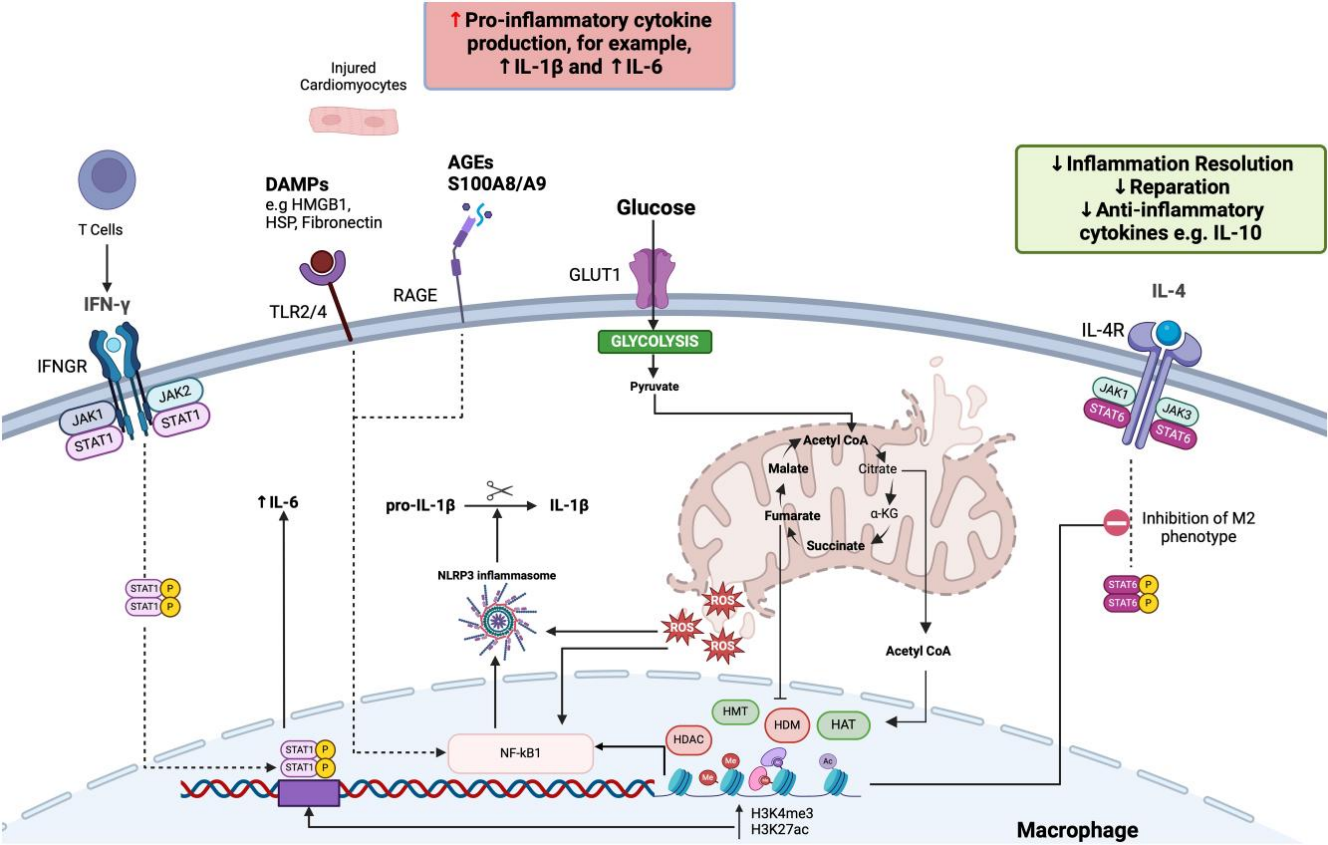
Cellular processes of inflammation relevant to acute MI in diabetes. After acute myocardial infarction (AMI), damaged cardiomyocytes release damage-associated molecular patterns (DAMPs), which activate pattern recognition receptors (PRRs) on macrophages including Toll-like Receptors (TLR) 2 and 4 and the receptor for advanced glycation end products (RAGE). This activation triggers nuclear factor kappa B (NFκB) signalling, subsequently activating the NOD-, LRR- and pyrin domain-containing protein 3 (NLRP3) inflammasome. Reactive oxygen species (ROS) contribute to NLRP3 inflammasome activation, culminating in the production of interleukin-1 beta (IL-1b). Furthermore, injured cardiomyocytes stimulate T cells to produce interferon-gamma (IFN-γ), which in turn activates the Janus kinase/signal-transducer and activator of transcription (JAK-STAT) pathway within macrophages, leading to the production of interleukin-6 (IL-6). Increased glucose entry into the cell induces metabolic reprogramming and modifies epigenetic enzymes such as histone methyltransferases (HMT), histone demethylases (HDM), histone acetyltransferases (HAT), and histone deacetylases (HDAC). This leads to key histone modifications, including histone 3 lysine 4 trimethylation (H3K4me3) and histone 3 lysine 27 acetylation (H3K27ac). These modifications alter chromatin accessibility at the IL-6 promoter, 'holding it open' to facilitate active transcription. Additionally, these epigenetic marks enhance NFκB activity. Moreover, these epigenetic changes inhibit anti-inflammatory pathways within macrophages, such as interleukin 4 (IL-4) signalling, consequently decreasing the production of anti-inflammatory cytokines such as interleukin 10 (IL-10). Ultimately, hyperglycaemia skews macrophage function away from an M2 anti-inflammatory phenotype towards a pro-inflammatory M1 phenotype. a-KG, a-ketoglutarate; GLUT1, glucose transporter-1; HMGB1, high mobility group protein B1; HSP, heat-shock proteins; IFNGR, interferon-gamma receptor; IL-4R, interleukin-4 receptor. Created with BioRender.com.

In animal models, hyperglycaemia drives myelopoiesis,^162^ which seems to be exacerbated by patterns of transient intermittent hyperglycaemia.^163,164^ Recently, we have identified a new phenomenon of Hyperglycaemia-Induced Trained Immunity (HITI) in which hyperglycaemia exacerbates classical inflammation and suppresses repair. Hyperglycaemia leads to epigenetic changes in haematopoietic stem cells that prime their progeny away from the M2 reparative phenotype towards the M1 pro-inflammatory phenotype. These modifications persist despite restoration to physiological levels of glucose.^165^ While this (M1/M2) dichotomy is an oversimplification, our findings suggest that HITI might influence processes of inflammation after MI and lead to changes that are not corrected by lowering glucose. In *Figure 2*, we suggest how DM may affect the inflammatory process in macrophages after MI.

As in peripheral wound healing, DM and HITI may impair infarct healing in the heart. Efficient wound healing requires the sequential infiltration of pro-inflammatory monocytes in the inflammatory phase followed by anti-inflammatory monocytes in the reparative phase. However, in wounds from diabetic mice, Ly6C^Hi^ monocytes (i) display a ‘second influx’ and infiltrate the wound in the reparative phase of wound healing and (ii) delay their transition to Ly6C^Lo^ monocytes.^166^ A similar, yet unexplored, process could be occurring in the heart. As previously mentioned, hyperglycaemia leads to a bias away from the M2 reparative phenotype. The infiltration of pro-inflammatory monocytes to the ischaemic myocardium could impair the resolution of inflammation and exacerbate the amount of injury. If these are pathologically important processes, one might expect infarct size to be larger in people with DM, but that was not apparent in the CMR studies discussed above, (which showed no significant difference in infarct size measured in the acute phase)^60,61^ though it is known that infarct size only reaches its final size after approximately 30 days.^167^ Hence, it is possible that infarct size was measured too early before it was possible to detect differences, especially if HITI delays infarct healing and inflammation resolution. Furthermore, current imaging techniques do not have the resolution to detect potentially important histological differences in the infarct healing process.

These diabetes-related effects on haematopoiesis and leucocyte function seem likely to influence the nature of leucocytes that are recruited to the remote myocardium. In human hearts, the presence of CCR2+ macrophages (derived from circulating monocytes) is associated with worse ventricular function and adverse remodelling.^168^ Monocytes biased towards pro-inflammatory processes might be recruited not only to the infarct zone, but also to the remote myocardium, which could partially explain why people with DM are more likely to develop HF after MI.^169,170^ Furthermore, recent evidence suggests that inflammatory cells can be a determinant of arrhythmia after MI.^171^

Systemically, hyperglycaemia increases the production of inflammatory cytokines (TNF-α, IL-6, and IL-18)^172^ and acute phase proteins.^173^ As previously mentioned, hs-CRP is strongly associated with residual risk of cardiovascular events, notably in people with DM.^129^ CRP production is driven by IL-6.^174^

Inhibition of IL-1β with the monoclonal antibody canakinumab lowers hs-CRP specifically in people with DM^175^ but subgroup analysis of the CANTOS trial did not show selective benefits for those with a diagnosis of T2D.^176^

Plausibly, and for the reasons discussed above, the presence of ‘nominal’ DM may be insufficiently specific to identify a subgroup of people with DM who might benefit from targeting active inflammation. At a cellular level, hyperglycaemia drives glycolysis which, in turn, leads to post-translational modifications in specific histones. Significantly these histone modifications alter chromatin accessibility at the IL-6 promoter, which is a cardinal feature of HITI.^165,177^ Effectively the promoter is ‘held open’ rendering the IL-6 gene primed for active transcription. Accordingly, people in whom T2D had led to HITI are likely to (i) have higher levels of IL-6 and hs-CRP and (ii) show particular therapeutic benefit to IL-6 inhibition.

## 8. Conclusions and future implications

The global prevalence of T2D is increasing rapidly with adverse implications for cardiovascular disease. Despite overall improvements in outcomes after MI, people with T2D have had persistently worse outcomes with higher rates of complications, re-infarction, and death. The contributory factors are complex and multiple and extend far beyond ‘anatomical’ considerations of the extent of angiographically apparent CAD and or myocardial infarct size. In particular, emerging strands of evidence point to a role for processes of inflammation and impaired inflammation resolution in T2D.

Epidemiological studies show that in people with T2D, in whom LDL-cholesterol is treated to contemporary targets, elevated high-sensitivity C-reactive protein is a superior predictor of outcome (compared with LDL-cholesterol). Recent data have shown a role for hyperglycaemia-driven myelopoiesis and epigenetically re-programmed bone marrow with adverse consequences of innate immune function. These alterations in innate immune function are of likely relevance to both atherosclerosis progression and response to acute MI.

All people with ‘diabetes’ are not susceptible to the same extent. The emerging complexity strongly suggests that in future, people will benefit from the description of disease *processes* that extend beyond the mere nominal diagnosis ‘diabetes’. In particular, better understanding and characterization of consequences for processes of inflammation and disordered homeostasis are likely to open new opportunities to target complications and improve cardiovascular outcomes of people with DM.
